# Synthesis and release behavior of layered double hydroxides–carbamazepine composites

**DOI:** 10.1038/s41598-021-00117-9

**Published:** 2021-10-18

**Authors:** Ma. F. Peralta, S. N. Mendieta, I. R. Scolari, G. E. Granero, M. E. Crivello

**Affiliations:** 1 Centro de Investigación y Tecnología Química – CONICET – Universidad Tecnológica Nacional, Regional Córdoba, Maestro López Esq. Cruz Roja Argentina, S/N, X5016ZAA, Córdoba, Argentina; 2 Unidad de Investigación y Desarrollo en Tecnología Farmacéutica – CONICET – Universidad Nacional de Córdoba, Córdoba, Argentina; 3 Departamento de Ciencias Farmacéuticas, Universidad Nacional de Córdoba, Córdoba, Argentina

**Keywords:** Materials science, Nanoscience and technology

## Abstract

Carbamazepine (CBZ) was incorporated into layered double hydroxides (LDH) to be used as a controlled drug system in solid tumors. CBZ has a formal charge of zero, so its incorporation in the anionic clay implies a challenge. Aiming to overcome this problem, CBZ was loaded into LDH with sodium cholate (SC), a surfactant with negative charge and, for comparison, without SC by the reconstruction method. Surprisingly, it was found that both resultant nanocomposites had similar CBZ encapsulation efficiency, around 75%, and the LDH-CBZ system without SC showed a better performance in relation to the release kinetics of CBZ in simulated body fluid (pH 7.4) and acetate buffer simulating the cellular cytoplasm (pH 4.8) than the system with SC. The CBZ dimensions were measured with Chem3D and, according to the basal spacing obtained from X-ray patterns, it can be arranged in the LDH-CBZ system as a monolayer with the long axis parallel to the LDH layers. Fourier transform infrared spectroscopy and solid state NMR measurements confirmed the presence of the drug, and thermogravimetric analyses showed an enhanced thermal stability for CBZ. These results have interesting implications since they increase the spectrum of LDH application as a controlled drug system to a large number of nonionic drugs, without the addition of other components.

## Introduction

Currently, cancer is the second cause of death in the world, which makes it one of the most important subjects of study in modern medicine. From the diverse types of the disease, solid tumors are the most frequent, with breast cancer in the first place (2.26 million cases in 2020), followed by lung (2.21 million cases in 2020), and colon and rectum (1.93 million cases in 2020)^1^. At present, one of the principal treatments against cancer is chemotherapy, which consists in the use of drugs that predominantly kill tumor cells. Even with the advances in the last decades, there is still ample room for improvement since it produces many side effects^2,3^.

Another serious obstacle for effective anticancer drugs is their low solubility in water and low bioavailability; most of them require a vehicle to be carried in the organism. In this sense, many drug administration systems have been developed; among them, polymeric nanoparticles, micelles and liposomes are the most outstanding^2,4^. Inorganic nanosystems, among which are the layered double hydroxides (LDH), are becoming strong competitors^5–7^. LDH are composed of metal cationic layers and interlamellar spaces filled with negative ions or anionic drugs^8–10^. These substrates have several advantages: defined properties for the controlled release of the drugs (stability and consequent drug protection during blood circulation (pH 7.4) and good endosomal escape and biodegradation, with consequent drug release, in the cellular cytoplasm (pH between 4 and 6)^11–13^), biocompatibility, efficient penetration of the cellular membrane^14,15^ and protective effect for the drug against temperature, light, humidity and oxidation^16,17^. Additionally, LDH have the advantage of being nano-sized, from 20 to 200 nm, which is a fundamental property for drug delivery systems intended for systemic administration.

Remarkably, there is not a commercial medication that uses LDH as a vehicle of the active principles yet. However, the necessity of new systems and the benefits of these carriers are indisputable, which leads to an urgent need to advance in the research of these inorganic nanosystems. Only the empty LDH, of the hydrotalcite type, is currently commercially available and it is used as antacid for the treatment of stomach diseases^18^.

Another challenge for modern medicine in the field of chemotherapy is to find novel anticancer drugs. With this objective, approved drugs against other kinds of diseases are being tested in different tumors. To date, the drugs studied in LDH are methotrexate, cisplatine, 5-fluorouracil and doxorubicin^19–21^. However, anticancer drugs without anionic charges or new molecules with potential anticancer activity have not been tested. This is important for the success in the search for new anticancer treatments, especially because the use of LDH can improve the antitumor activity of the new molecules^19,20,22,23^. Carbamazepine (CBZ) is an anticonvulsant drug with a formal charge of zero. It has shown efficacy against some solid cancer cell lines, such as human breast cancer^24,25^ and human colon adenocarcinoma^26^. However, CBZ also produces alterations in red blood cells^27^.

CBZ is poorly soluble in water, and many authors have increased the solubility through its complexation with D-gluconolactone^28^, polymers^29^, cyclodextrins^30^ or surfactants such as sodium lauryl sulfate and Tween 80^31^. Few works studied the incorporation of CBZ in different matrices for water remediation; this includes adsorption of CBZ on silica-based porous materials^32^, on granular carbon nanotubes /alumina hybrid adsorbent^33^, and on hematite nanoparticles^34^. To the best of our knowledge, no studies have been performed with CBZ incorporated in LDH, and no systems have been designed to increase CBZ anticancer activity. The incorporation of CBZ in an LDH system can increase its activity since these nanoclays are excellent intracellular delivery carriers^18^. The internalization of LDH into the cells can be mediated mainly via clathrin endocytosis when the nanoparticles range between 50 and 200 nm in diameter^35–38^. This endocytosis is enhanced by the positive surface charges of the nanoclay. Once in the cytoplasm of the cells, the composites are released from the endosomes, the LDH degrade due to the acidic pH and the drug can quickly produce its anticancer effect. Moreover, LDH are biodegradable, which implies that they are rapidly eliminated from the body without leaving traces in other organs^8^. Finally, these substrates have very low toxicity, especially when compared to silica, carbon nanotubes and iron oxides; LDH with a size of 100–200 nm produce less inflammation, membrane damage, and cell proliferation^8^.

Sodium cholate (SC) is a bile salt surfactant with negative charge that forms micelles at the critical micelle concentration. In the process, the drugs that are in the medium can be encapsulated into the micelles. SC can be used to either enhance the solubility of drugs by offering a hydrophilic and a hydrophobic face^39^ or give negative charge^40,41^.

In this work, we propose the use of a drug with potential anticancer activity that is not currently used as cytotoxic, with the increase of its activity and its selectivity by incorporating it in an inorganic solid, taking advantage of the LDH capability to avoid drug action during circulation and enhanced tumor cell penetration by clathrin endocytosis. As most of these drugs have no formal charge, a functionalization of the molecule could be needed for their incorporation in the substrate. In this way, CBZ was incorporated into LDH composed of Mg–Al–NO_3_ with SC by using two methods: ionic exchange and reconstruction. The reconstruction method was also performed by loading the CBZ without SC in order to study a possible incorporation of the drug into LDH through weak interactions. Different techniques were applied to characterize the systems and to determine their efficacy as drug carriers.

## Results

**Figure 1:**
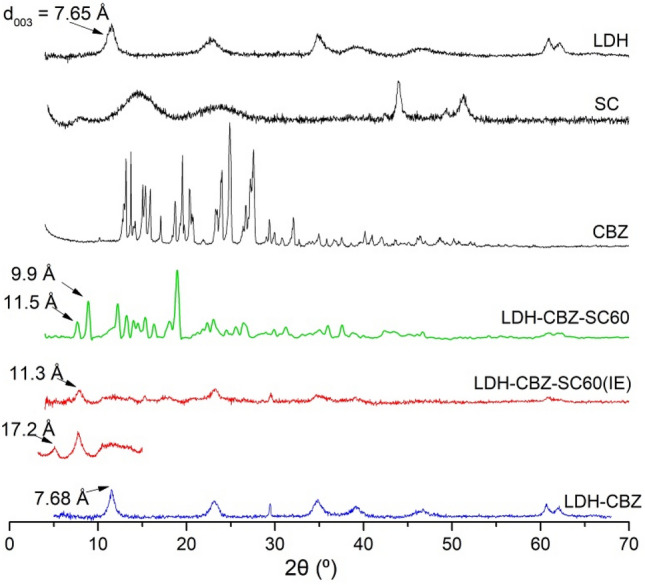
Powder X-ray diffraction patterns of LDH, CBZ, SC, and composites.

**Figure 2:**
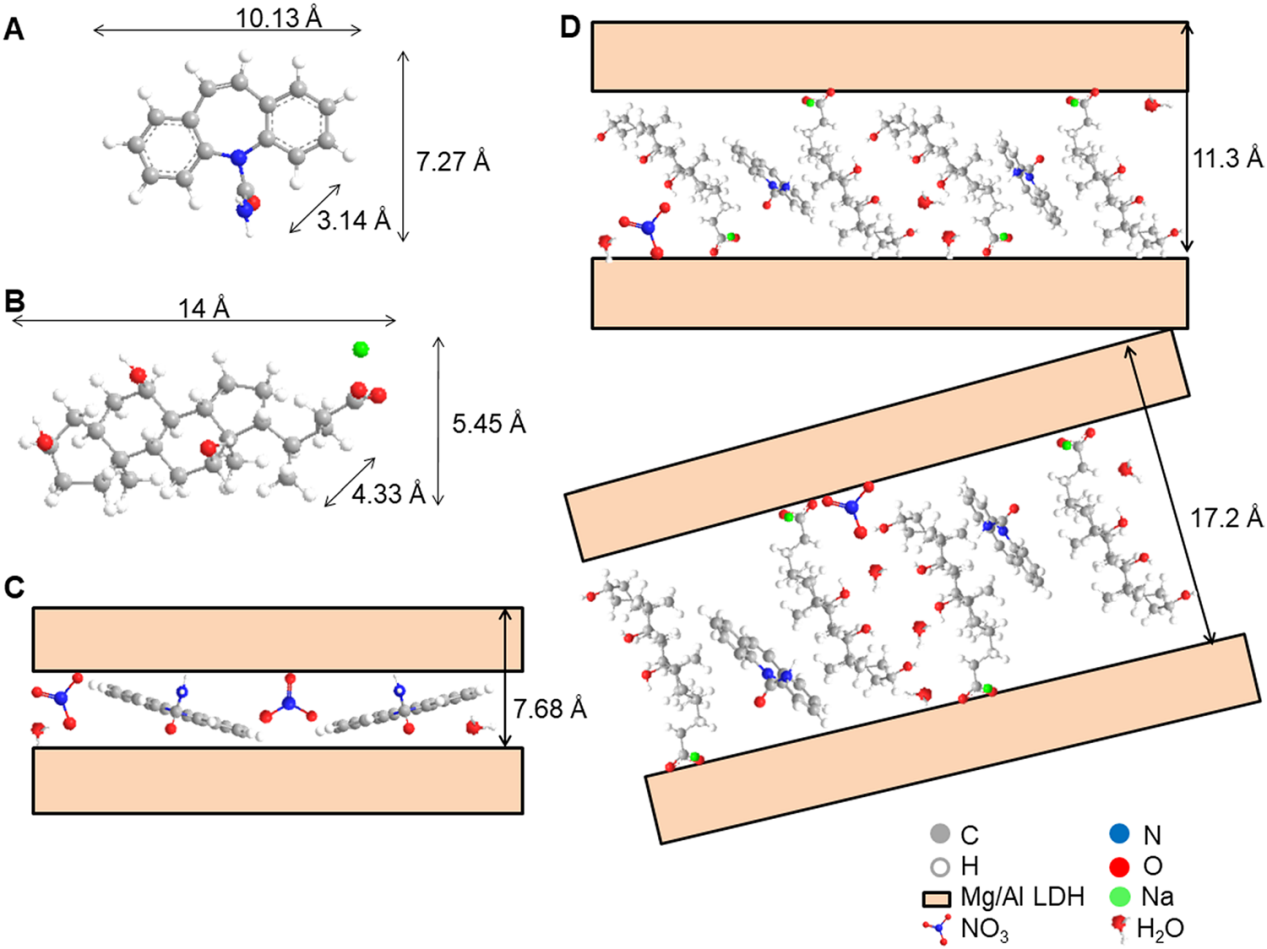
**(a)** CBZ dimensions calculated with Chem3D. (**b)** SC dimensions calculated with Chem3D. (**c)** Probable CBZ orientation in LDH-CBZ. (**d)** Probable CBZ and SC orientation in LDH-CBZ-SC60(IE).

The XRD patterns of simple LDH (solid host) and CBZ intercalated, LDH-CBZ, with or without SC60 (reconstruction and ion exchange method) are shown in Fig. 1. The diffraction patterns of LDH and LDH-CBZ samples show quite sharp (0 0 l) and (1 1 l) peaks, typical of a well-crystallized material. The basal spacing (d_003_) corresponding to NO_3_^-^ ions was 7.65 Å; this value was very similar to that of the LDH-CBZ (7.68 Å). CBZ dimensions were measured with Chem3D 18.0 (Perkin Elmer) and are shown in Fig. 2a; Fig. 2c shows the way that CBZ might be accommodated into the LDH, taking into account that no superficial drug appeared in the diffractogram.

In the samples containing SC, two peaks were observed, corresponding to basal spacings (d_003_) of 11.5–9.9 Å and 17.2–11.3 Å for LDH-CBZ-SC60 and LDH-CBZ-SC60(IE), respectively (Fig. 1). On the other hand, LDH-CBZ-SC60 and LDH-CBZ-SC60(IE) samples exhibited large and asymmetric (0 1 l) reflections, indicating stacking faults. The diffraction pattern of LDH-CBZ-SC60 sample also shows some peaks characteristic of pure CBZ (Fig. 1, 2θ = 13.1°, 15.3°, 18.8°, 23.2°, 24.7°, and 26.6°^42–44^), which indicates that some drug remained at the surface of the LDH. Taking into account the size of SC molecules, measured with Chem3D and displayed in Fig. 2b, we propose a probable organization of CBZ/SC in the LDH as inclined monolayers with CBZ molecules located near the hydrophobic face of SC (Fig. 2d).

**Figure 3:**
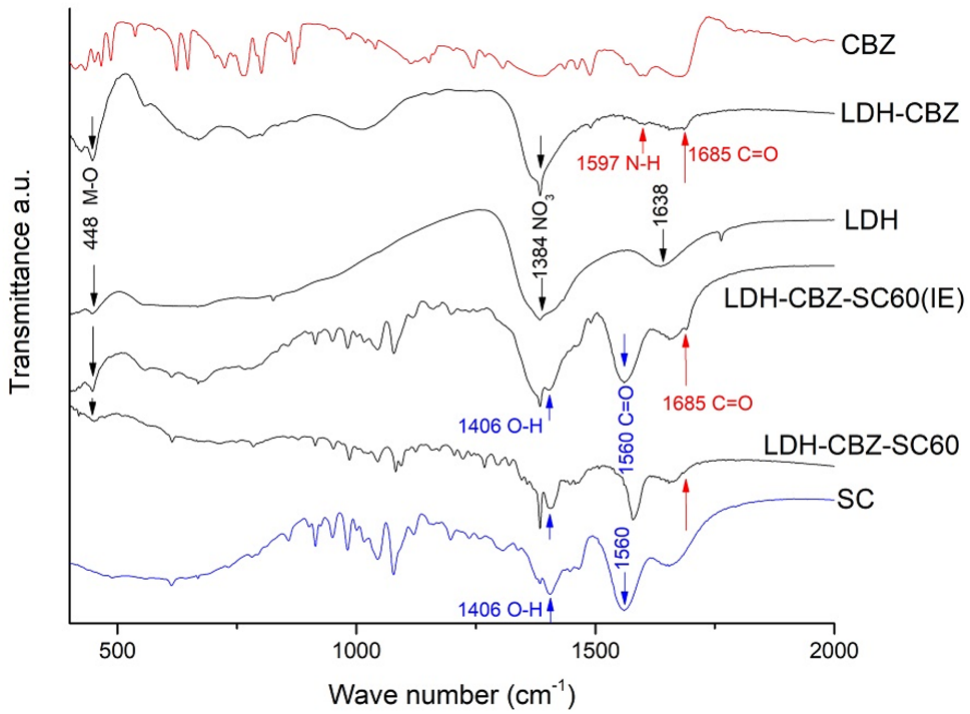
FTIR spectra of CBZ, SC, LDH and CBZ-LDH composites.

The characteristic transmittance peaks of CBZ, at 1597 cm^−1^ (N–H deformation) and 1685 cm^−1^ (C=O stretching)^45^, appear in the FTIR spectrum of LDH-CBZ (Fig. 3), indicating that CBZ molecules were loaded into LDH. Absorption at 1384 cm^−1^, present in all samples, can be assigned to the *v*_3_ vibration of NO_3_^−^, which is also in the interlayers. The band at 1638 cm^−1^, due to the bending mode of water molecules, disappears in the LDH-CBZ spectra. The peak at 448 cm^−1^ is attributed to metal-O lattice vibrations. The spectra of the samples containing SC showed their characteristic peaks at 1406 cm^−1^ (O–H vibrations) and 1685 cm^−1^ (C=O stretching)^46^. These spectra were difficult to analyze since the surfactant bands are wide and strong and overlap the peaks of the CBZ (Fig. 3); however, the peak at 1685 cm^−1^ (C=O stretching) is detectable, which shows the presence of the drug in the composites. This absorption peak is weak for two reasons: the movement of CBZ in the composites is slower in comparison with the free drug, which produces the low IR absorption of the drug; and the concentration of the drug used in the synthesis is low (5% of the total components)^46^.

**Figure 4:**
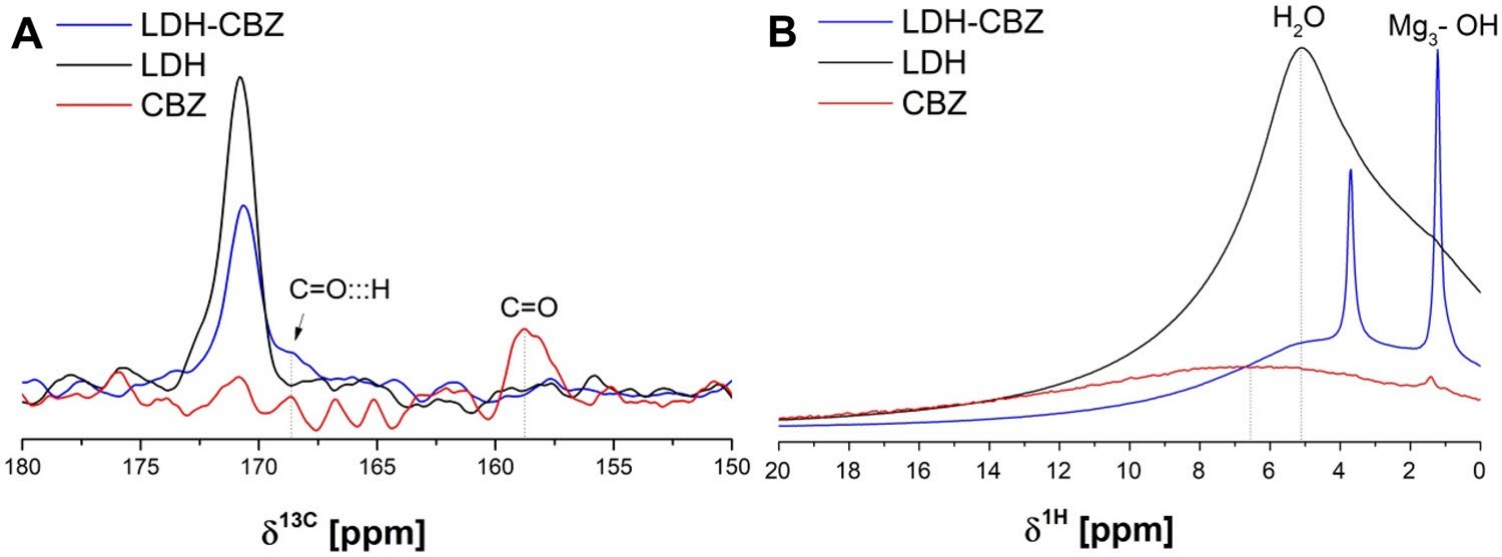
**(a**) ^13^C and (**b**) ^1^H solid state NMR spectra.

The molecular interactions through the C=O group of CBZ and LDH were studied using ^13^C NMR (Fig. 4a). The noise/signal ratio was quite high in all the spectra; however, the C=O chemical shift of the pure CBZ was around 158 ppm, in agreement with Ishizuka and Zhou^43,47^. The LDH-CBZ spectrum showed a peak shifted to higher values, at approximately 168 ppm, associated with hydrogen bonding^48^, which was broadened due to the interaction with the OH layer or water molecules of the interlayer. The resonance at 170 ppm in the LDH and LDH-CBZ samples could be ascribed to interlayer carbonate species, but by FTIR spectroscopy it is not possible to detect the presence of carbonate; then it must be present only in a minor amount and it is probably adsorbed on the external surface of the solid as a result of carbonation by atmospheric CO_2_^49^.

In the ^1^H NMR spectra of pure CBZ (Fig. 4b), the protons of the aromatic groups appeared at 6.5 ppm^47^. The resonance peak at 5.1 ppm in the LDH sample represents water occluded in the interlayers^50–52^; the intense water resonance reflects large water content in the intergallery of the empty LDH that significantly decreases with the incorporation of CBZ. The peaks at 1.2–1.3 ppm correspond to Mg_3_-OH interactions between the metals of the hydroxide sheets and the water of the interlayer, while the resonance at 3.7 ppm, which is visible only after the incorporation of CBZ, corresponds to MgAl_2_-OH interactions. The higher intensity of the peak at 1.2 ppm with respect to that at 3.7 ppm is due to the metals ratio used in the synthesis (Mg:Al ratio 2:1). This trend also indicates that the Al and Mg cations must be ordered mainly in the metal hydroxide sheets.

**Figure 5:**
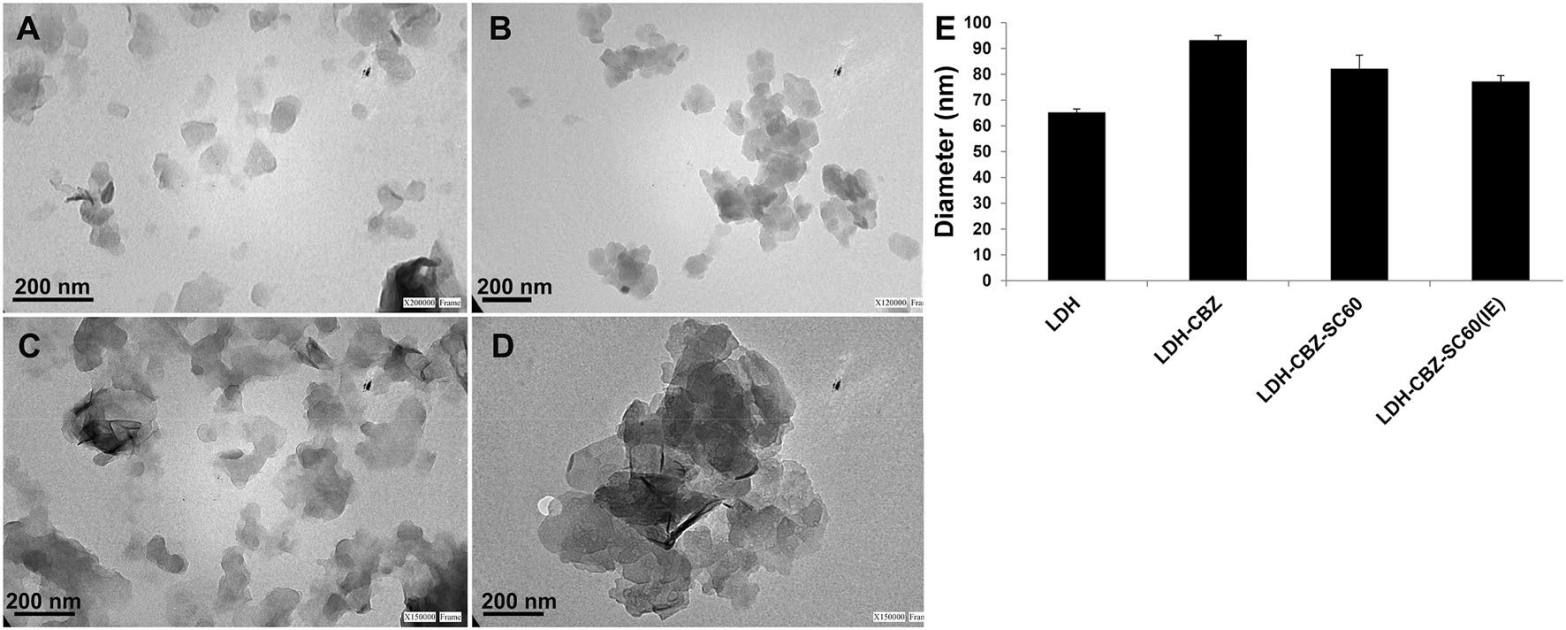
TEM images of (**a**) pristine LDH, (**b**) LDH-CBZ, (**c**) LDH-CBZ-SC60(IE), and (**d**) LDH-CBZ-SC60. (**e**) Diameter of LDH in different samples measured from TEM images.

TEM images (Fig. 5a–d) shows the laminar structure of LDH in all the samples, with the typical thin, plate-like shape and dimensions below 100 nm (Fig. 5e). The addition of SC and CBZ did not change the dimension of the crystals although they tended to agglomerate them. In the samples with SC, the ion exchange method seems better than reconstruction, since aggregates are smaller (Fig. 5c,d respectively).

**Figure 6:**
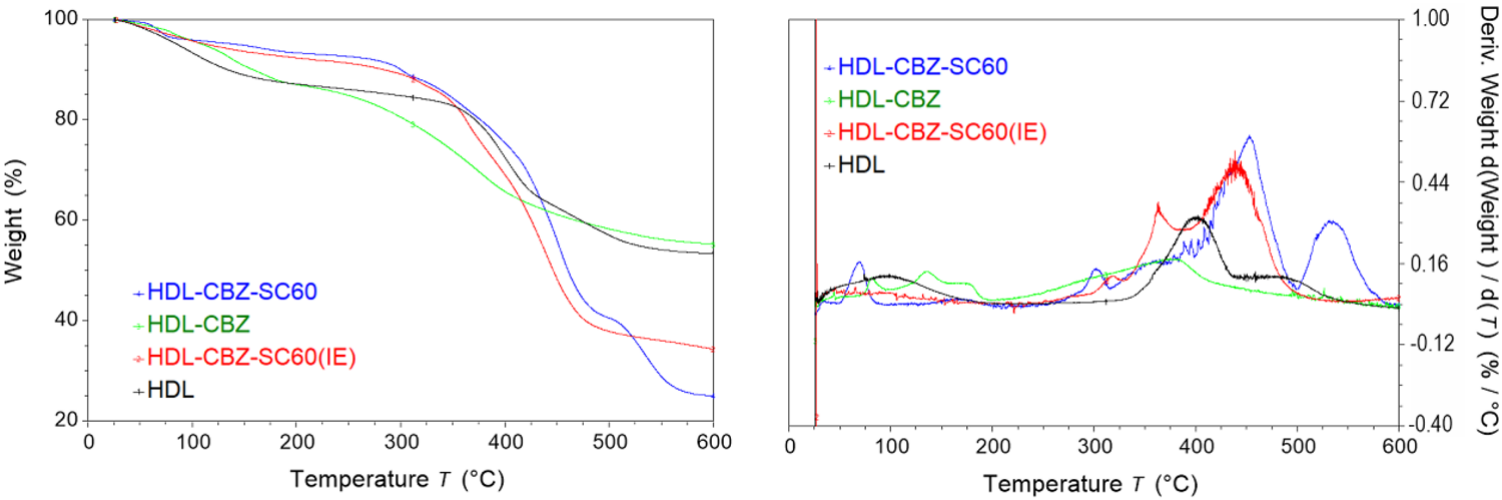
TG and DTG curves of composites.

Thermal behavior was determined by TG and DTG (Fig. 6). For LDH, the host solid, one weight loss stage can be observed at low temperatures. The maximum peak at 100 °C is due to physically adsorbed water, and this stage ends at 200 °C, which corresponds to the loss of interlayer water. The whole process represented a 13% weight lost, which indicates low hydration of the host solid. Finally, above 350 °C, the dehydroxylation of the brucite-like sheets and the loss of interlayer ions indicate that the layer has disappeared. The TG curves of the samples containing SC depict a similar weight loss of around 70%. In the LDH-CBZ sample, the total weight loss was only 45%; this difference is due to the absence of SC. In the sample synthesized by reconstruction, DTG analyses revealed that the first thermal event, which occurred between 70 and 80 °C, corresponds to CBZ dehydration^53,54^; the transitions observed up to 170 °C are associated with the removal of interlayer water. Finally, the last transition corresponds to SC decomposition^55^. In the LDH-CBZ-SC60(IE) sample it is not possible to observe a high loss of physically adsorbed water, due to the low moisture content present in the LDH host.

Table 1 shows the results of the CBZ loading into the LDH from the different synthesis methods. Encapsulation efficiency (EE) was high in all the samples; it was similar for LDH-CBZ and LDH-CBZ-SC60 (75%), and its value increased to 95% in LDH-CBZ0-SC60(IE).

**Table 1:** CBZ loading and encapsulation efficiency in the different composites.

	CBZ loading (%)	Encapsulation efficiency (EE) (%)
LDH-CBZ	5.3 ± 0.6	75
LDH-CBZ-SC60	1.6 ± 0.0	75
LDH-CBZ-SC60(IE)	1.8 ± 0.2	95

**Figure 7:**
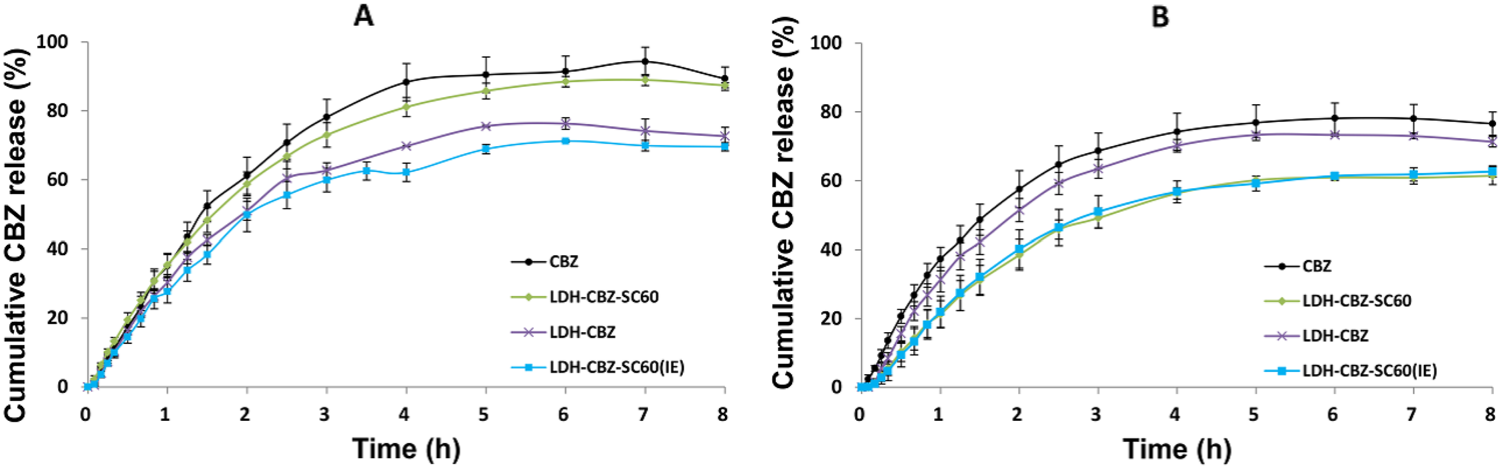
In vitro CBZ release profiles in (**a)** simulated body fluid (SBF), pH 7.4 and (**b)** acetate buffer, pH 4.8. CBZ solution in the respective mediums was used as reference (mean ± SD, n = 3).

The release profiles of CBZ, pure and from the different composites, are shown in Fig. 7. The patterns at pH 7.4 and 4.8 cannot be directly compared because CBZ behaves differently in the two mediums; the pure drug dissolves better and goes faster to the receptor medium at pH 7.4 than at 4.8. The statistical analysis of these results was done by comparing the CBZ release profiles of the different systems with that one of the free drug. Table 2 shows the F1 and F2 factors, where F1 < 15 and F2 > 50 imply the release profiles are equals or similar.

**Table 2:** Statistical analysis of CBZ release profiles. Comparisons were made for each system developed with respect to the free drug.

	SBF (pH 7.4)			Acetate buffer (pH 4.8)		
	F1	F2	Interpretation	F1	F2	Interpretation
LDH-CBZ	17.2	48.2	Different curves	10.7	64.9	Similar curves
LDH-CBZ-SC60	5.5	72.9	Similar curves	30.5	41.5	Different curves
LDH-CBZ-SC60(IE)	22.9	42.3	Different curves	29.9	42.1	Different curves

The diffusion release mechanism of CBZ from LDH was verified by fitting for linearity between − ln (1 − Q_t_/Q_0_) and t^0.65^, according to Bhaskar equations (Table 3). CBZ may have been released from LDH at pH 7.4 by controlled diffusion, since the Bhaskar method showed linearity with an R^2^ of 0.9953. The correlation coefficient for the experiment at pH 4.8 was also good, although a bit smaller: 0.9937. CBZ can also be released from samples with SC by diffusion (Table 4).

**Table 3:** Equations for fitting the data of the CBZ release.

Model	Equation	References
Bhaskar	− ln (1 − Q_t_/Q_0_) = 1.59 (6/d_P_)^1.3^ D^0.65^ t^0.65^	^56,57^
Pseudo-first-order	ln (Q_e_ − Q_t_) = ln Q_e_ − K_1_ t	^58,59^
Pseudo-second-order	t/Q_t_ = 1/K_2_ Q_e_^2^ + t/Q_e_	^59,60^

*t* time, *Q*_*0*_ drug content at time t = 0, *Q*_*t*_ drug content at any time, *Q*_*e*_ drug content at equilibrium, *d*_*p*_ particle diameter, *D* diffusivity.

**Table 4:** Correlation coefficient (R^2^) obtained by fitting the data of CBZ release into SBF (pH 7.4) and acetate buffer (pH 4.8).

		Bhaskar	Parabolic diffusion	Pseudo-first-order		Pseudo-second-order
					K_1_ (min^−1^)	
LDH-CBZ	pH 7.4	0.9953	0.5993	0.9950	0.6404	0.9349
	pH 4.8	0.9937	0.3500	0.9845	0.7525	0.9163
LDH-CBZ-SC60	pH 7.4	0.9896	0.7732	0.9852	0.6884	0.9815
	pH 4.8	0.9940	0.1880	0.9795	0.6540	0.4043
LDH-CBZ-SC60(IE)	pH 7.4	0.9488	0.6236	0.9881	0.6364	0.9373
	pH 4.8	0.9876	0.0548	0.9720	0.7551	0.1614

**Figure 8:**
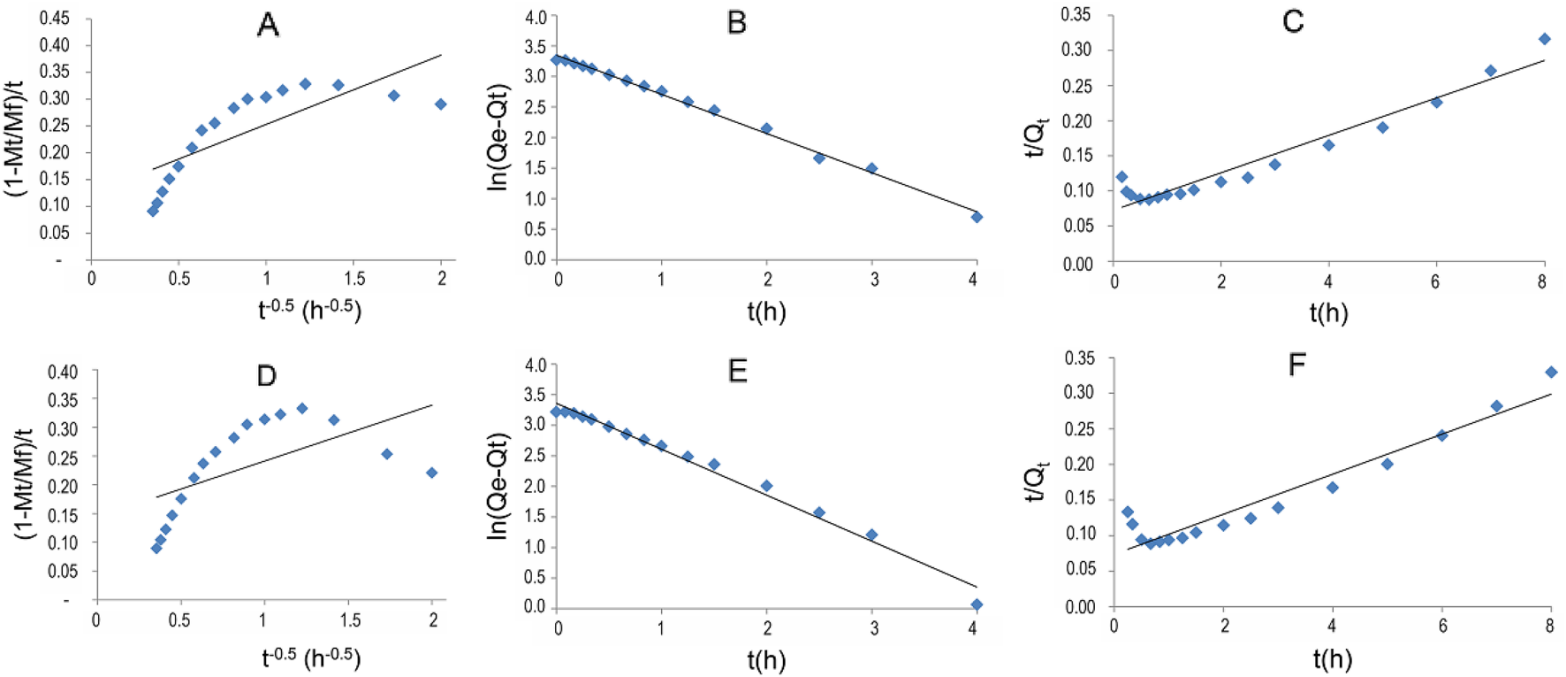
Fitting of the data of CBZ release from LDH-CBZ composite to parabolic diffusion (**a**,**d**), pseudo-first (**b**,**e**) and pseudo-second (**c**,**f**) order kinetics for SBF, pH 7.4 **(a–c)** and acetate buffer, pH 4.8 **(d–f)**.

The pseudo-first-order kinetic model was evaluated by fitting for linearity between ln (Q_e_ − Q_t_) and t, according to the model equation, shown in Table 3. The rate constant K_1_ was obtained from the slope of the linear plot. The pseudo-second-order kinetic model was evaluated by testing for linearity between t/Q_t_ and t (Table 3). Results are shown in Table 4 and Fig. 8; the pseudo-first-order model was the most satisfactory for describing the release kinetic processes of CBZ from the LDH-CBZ composite, with an R^2^ of 0.995 and a K_1_ of 0.64 min^−1^ at pH 7.4 and an R^2^ of 0.985 and K_1_ of 0.75 min^−1^ at pH 4.8. The samples with SC also fitted better the pseudo-first-order kinetic model (Table 4) (R^2^ between 0.969 and 0.988) than the other one.

## Discussion

CBZ is a neutral molecule that has shown activity against some cancer cell lines. The incorporation of the drug into LDH could avoid its side effects during blood circulation and allow its activity at the target sites. LDH are clays composed of cationic layers stabilized with anionic molecules that locate between them. SC, an anionic surfactant, was used to facilitate CBZ incorporation into the LDH interlayers, and the free drug was also incorporated into the LDH without SC for comparison purposes.

The synthesized LDH had a well-crystallized structure. The low value observed in the interlaminar distance may be due to the low hydration that the material presents. Other authors, who suggest that the nitrate anion accommodates itself in a parallel way to the brucite layer without widening due to the few water molecules that surround it, obtained similar data. Iyi et al. reported c values between 21.6 and 22.8 Å for nitrate anions^61,62^.

The structure and basal spacing of the LDH containing CBZ (without SC) were very similar to that of the LDH. The very small increase in the d_003_, can be due to the small size of the molecule (molecular size = 236 Da)^63,64^, which allows it to stay in the interlayer without changing its initial distance. The interlayer domains contain not only anions but also water and neutral molecules^65^, and the nonionic CBZ molecules perhaps could be stabilized in the gallery of LDH because of hydrogen bonds between H of OH (from brucite-type layers) and O or N of the CBZ molecules intercalated. These results agree with those of Dong et al.^66^, who reported that the incorporation of camptothecin, a molecule similar and slightly larger than CBZ, produced only a small increase in the interlayer space. The authors propose that, in the LDH-CBZ system without SC, the molecules arrange as monolayers with the long axis parallel to the LDH layers.

Despite low percentage of drug used, it is possible to observe the peaks corresponding to CBZ in the FTIR and NMR spectra of the LDH-CBZ composites and infer its presence in the interlayers. The drug encapsulation efficiency was 75%, and by X-ray diffraction superficial CBZ was not observed, unlike the LDH-CBZ-SC60 sample. In the solid-state ^1^H NMR study, the concentration of interlayer water and hydroxyl groups results in large ^1^H homonuclear dipolar couplings, which broadens the resonances and prevents the identification of chemically distinct ^1^H environments^50^; in the LDH spectrum, where a broad peak appears with a maximum at 5.1 ppm (water molecules), that phenomenon occurred. The incorporation of CBZ replaces some water and allows the definition of the peaks at 3.7 and 1.2 ppm corresponding to MgAl_2_-OH and Mg_3_-OH respectively; this is in accordance with the decrease in the peak at 5.1 ppm in the LDH-CBZ sample and the elimination of the peak at 1638 cm^-1^ in the FTIR spectrum. The higher definition of the peaks in the LDH-CBZ sample with respect to the empty LDH is then due to the presence of CBZ molecules in the interlamellar space, which replaces the interaction between water and metal hydroxides. Moreover, the ^13^C spectrum of LDH-CBZ sample shows the interaction between the C=O group of CBZ and the OH groups of the LDH layers and the remaining water molecules of the interlayer.

In the samples containing SC, the intensity of XRD peaks decreased in comparison with the LDH-host, indicating some reductions in crystallinity following the intercalation of SC and CBZ. Two basal planes were observed, which indicates different orientations of the CBZ-SC molecules in the LDH. The interlaminar distance observed is less than the size calculated for the SC (between 10–11 Å and 14 Å respectively). This value assumes a slightly sloped monolaminar arrangement for the SC-CBZ molecules. SC molecules intercalated in the LDH alone or joined to CBZ cannot be distinguished.

The presence of the surfactant provides the interlaminar zone with a hydrophobic environment conductive to host the nonionic CBZ. CBZ is a hydrophobic molecule that accommodates near the hydrophobic face of SC and interacts with the surfactant through this face; a slight interaction such as hydrogen bonds between the amino groups of CBZ and hydroxyls of SC would not be ruled out. The SC carboxylate group interacts with the brucite layer through electrostatic forces^67^. A similar behavior was observed in the solid obtained by ion exchange. Larger basal planes were obtained (17 Å), suggesting that SC-CBZ molecules fit into a tilted monolayer arrangement.

According to literature, the decomposition of pure CBZ begins at 200 °C^53,54^; in DTG diagrams the decomposition is not observed until 300 °C, which can be associated with the superficial CBZ observed in LDH-CBZ-SC60 by XRD. From 350 °C, the decomposition of the sheets begins; the dihydroxylation of the lamella, decomposition and combustion of the intercalated CBZ occur together. The thermal decomposition temperature of the CBZ was strongly affected by the host layer. These results confirmed the enhanced thermal stability of the drug.

The CBZ loading into the LDH, i.e., the percentage of CBZ in the system, was higher in LDH-CBZ than in the samples with SC because of the fewer components of the synthesis. The drug encapsulation efficiency was high (75%) and the same result was obtained with the systems with and without SC when the reconstruction method was used; this indicates that it is not necessary to incorporate SC together with CBZ into the LDH. The encapsulation was higher (95%) in the sample with SC synthesized by ion exchange, but this method of synthesis cannot be applied in the case of the uncharged free drug; the basic principle of this synthesis method is that anionic molecules exchange with the anions located in the interlayers during agitation; when there is no other anionic molecules around the LDH, the anions remain in the interlayer stabilizing the cationic layers and neutral molecules are not able to enter into the LDH.

Assays of in vitro drug release were done in Franz Cells with simulated body fluid (pH 7.4) and acetate buffer (pH 4.8) in order to simulate and analyze the protective role of the nanosystem developed in the blood and the drug release inside the tumor cells respectively. Compared with the free drug behavior in the Franz Cells, LDH-CBZ was the only system able to transport the drug at the pH value of the blood (pH 7.4) and release it at the pH value of the cell cytoplasm (pH 4.8). This was corroborated with the F1 and F2 factors, which statistically indicated that the release patterns of CBZ and LDH-CBZ were different at pH 7.4 and similar at pH 4.8. The good three-dimensional structure obtained, observed by XRD, was able to control the drug diffusion in the matrix at pH 7.4, and at acid pH it was destroyed, releasing CBZ in the medium. In the case of LDH-CBZ-SC60, the presence of CBZ at the surface of the LDH can explain the fast release of the drug at pH 7.4. A controlled release of CBZ was obtained with the system synthesized by anion exchange (LDH-CBZ-SC60(IE)) at pH 7.4 due to the predominant electrostatic interaction between the anionic SC and the brucite layer; however, the drug was not released at pH 4.8, same that the system obtained with the reconstruction method. The presence of SC could stabilize the system, so the LDH dissolve more slowly at acidic pH, and the hydrophobic face of SC attracts the CBZ molecules more than the aqueous medium present in the receptor compartment of Franz Cells. This behavior results in less release of CBZ. The release mechanism of CBZ from LDH, studied by Bhaskar method, seems to be a controlled diffusion in all the samples. At acidic pH, diffusion is also the principal process for CBZ release, and LDH dissolve but slowly. CBZ can also be released from samples with SC by diffusion, although, as described above, not efficiently.

The kinetics of drug release from LDH composites is usually described with pseudo-first and pseudo-second-order equations, or by parabolic diffusion^13,66,68^. The pseudo-first-order model was the most satisfactory in our systems. K_1_ values confirmed the faster release of CBZ at acidic than at neutral pH in LDH-CBZ samples. In the samples with SC, K_1_ values also confirmed the results discussed previously with CBZ release patterns. We also fitted the data with Ritger and Peppas, modified Freundlich, K-K and Higuchi models; however, their correlation coefficients were not sufficiently good.

## Conclusion

Results suggest that not only ionic bonds are involved in the structure of LDH but other types of interactions also take place, such as hydrogen bonds. This is why these systems are suitable not only for loading anionic molecules but are also promising carriers of drugs with different charges, especially for the numerous neutral anticancer ones. CBZ molecules could incorporate into the LDH when the reconstruction method was used, without the need of surfactant. This system presented an enhanced thermal stability for CBZ and a good drug loading with acceptable encapsulation efficiency. Compared to the free drug behavior, it was also able to transport CBZ with high stability at pH of 7.4, and to release it rapidly at pH 4.8, making it available inside the cell to exert its therapeutic effect. Thus this composite system may be a promising drug carrier for systemic administration of drugs targeted to intracellular sites, such as anticancer ones.

## Materials and methods

### Materials

Carbamazepine (USP grade, Parafarm) was provided by Saporiti, Argentina. Sodium cholate and Al(NO_3_)_2_·9H_2_O (98%) were purchase from Sigma Aldrich, St. Louis, USA. Mg(NO_3_)_2_·6H_2_O (98%) and NaOH (97%) were supplied by Biopack and ethanol (absolute grade) by Sintorgan®.

### Synthesis of composites

LDH were prepared by co-precipitation from Mg and Al nitrates. Two aqueous solutions of Mg(NO_3_)_2_·6H_2_O (0.6 M) and Al(NO_3_)_2_·9H_2_O (0.3 M) were prepared in 25 mL of decarbonated water, with a ratio of M^2+^/M^3+^  = 2. The solutions were added to 20 mL of decarbonated water at a rate of 1 mL/min, under permanent magnetic stirring. The coprecipitation was carried out at 70 °C under nitrogen atmosphere, to avoid the incorporation of CO_2_. The pH was maintained at 10.0 ± 0.2 by adding NaOH 2 M. The resulting suspension was stirred for 48 h under nitrogen atmosphere. The product was centrifuged and washed with decarbonated water until pH 7, and finally dried at room temperature. For the reconstruction method, LDH were calcined at 450 °C for 9 h and stored at 120 °C.

The LDH-CBZ sample was prepared by the reconstruction method. CBZ (1 mg) was dissolved in ethanol (1 mL); 16 mg of calcined LDH and 18 mL of decarbonated water were added to the solution, and the mixture was stirred for 48 h at 40 °C, in a nitrogen atmosphere. The solvents were finally evaporated in a rotary evaporator at 60 °C, and the resultant powder was recovered.

For LDH-CBZ-SC samples, CBZ (1 mg) was dissolved in ethanol (1 mL); SC (60 mg) and decarbonated water (8 mL) were added to the solution and the mixture was stirred for 10 min under a nitrogen atmosphere in order to allow ethanol evaporation. The amount of water was related to the amount of SC, in order to maintain the critical micelle concentration. Then, 16 mg of calcined LDH and 1 mL of water were added to the suspension, and the mixture was stirred for 48 h at 40 °C, in a nitrogen atmosphere. The solvents were finally evaporated in a rotary evaporator at 60 °C, and the resultant powder was recovered. Samples were called LDH-CBZ-SC60.

For comparison, LDH-CBZ-SC60(IE) was prepared by ion exchange method. The process was the same as that previously described, but LHD was incorporated in the original slurry (1 mL) instead of calcined.

### Characterization

Powder X-ray diffraction (XRD) patterns were collected on a X’Pert Pro-PANalytical diffractometer using Cu Kα radiation (λ = 1.54 Å) at a scan rate of 3°/min in 2θ and step size 0.02° in a scan range between 2° and 15° and at a step time of 4.25 s and step size 0.026° in 2θ, continuous, in a scan range between 4° and 70°.

Fourier transform infrared (FTIR) spectra were recorded on a Nicolet iS10 spectrometer at room temperature. The samples were pressed into a disc at 4 tons with KBr. The spectrum of each sample was recorded by accumulating 48 scans at 2 and 4 cm^−1^ resolution, between 400 and 4000 cm^−1^.

High-resolution ^13^C and ^1^H solid-state spectra for all the samples were recorded using a CP-MAS pulse sequence (cross polarization and magic angle spinning) with proton decoupling during acquisition. All the solid-state NMR experiments were performed at room temperature in a 7 T Bruker Avance II-300 spectrometer equipped with a 4-mm MAS probe. The operating frequency for protons and carbons was 300.13 and 75.46 MHz, respectively. Glycine was used as an external reference for the ^13^C spectra and to set the Hartmann-Hahn matching condition in the cross-polarization experiments. Adamantane was used as an external reference for the ^1^H spectra. The recycling time was 7 s for CBZ, 3.5 s for LDH-CBZ, and 3 s for LDH sample. Contact time during CP was 2 ms. The spinning rate for all the samples was 10 kHz.

Transmission electron microscopy (TEM) was performed in a JEOL JEM EXII 1200 microscope, operated at 80 kV; 0.5 mg of sample was dispersed in 4 mL of deionized water and sonicated for 30 min. A copper carbon grid was deposited on a small drop of the dispersion for 30 s and then allowed to dry. From TEM images, the diameter of each LDH was determined in two perpendicular directions using Fiji ImageJ software. In at least 4 images of each sample, all the well visible LDH were measured.

Thermogravimetric analysis (TG) was performed by means of an automatic thermal analyzer (TA Instrument, Discovery series). Thermal analyses were conducted at a scanning rate of 10 °C/min from 25 to 600 °C. The first derivative of TG (DTG) was determined with TRIOS TA Instrument software.

### Carbamazepine encapsulation efficiency

The loading amount of CBZ in the LDH was determined with a Jasco V-650 UV–Visible spectrophotometer. A known amount of the system was placed in a 5 mL volumetric flask and 2.5 mL of HCl 1 M solution was added to dissolve the inorganic layers. Ethanol was added to complete the final volume. The concentration of CBZ in the solution was determined at 284 nm using a calibration curve of CBZ in HCl:ethanol 1:1 v:v. The final value was an average of three independent samples. The CBZ loading was obtained according to the concentration of CBZ in the solution and the weight of the composite sample (Eq. 1).

1 $$Drug \; loading (\%) =\frac{amount \; of \; drug \times 100}{composite \; weight}$$

In addition, the drug encapsulation efficiency (EE) was calculated with Eq. (2)^69,70^, where actual loading refers to the loading amount of CBZ obtained from the experimental process.

2 $$EE (\%) =\frac{actual \; loading \times 100}{theoretical \; loading}$$

### In vitro carbamazepine release

The release of CBZ from LDH into simulated body fluid (SBF, pH 7.4, prepared as described by Cuello et al.^71^) and acetate buffer (pH 4.8) was achieved in vertical Franz cells under sink conditions (10% saturation). Each sample (equivalent to 40 µg of CBZ) was dispersed in 1 mL of the medium solution and added to the donor compartment. The receptor was filled with 9 mL of medium solution and maintained at 37 °C under agitation. Cellulose membrane of 14 kDa (Sigma-Aldrich, USA) was used to separate both compartments. Then, 1 mL of sample was taken from the receptor medium at different times and replaced by fresh medium. The accumulated amount of CBZ released was measured in the UV–Visible spectrophotometer (Agilent Technologies Cary 60 UV–Vis®) at 284 nm. Calibration curves in the respective mediums were used for CBZ concentration measurements.

From CBZ release patterns, molecule diffusion control was studied with the Bhaskar method^56,57^.

Release kinetics were studied with pseudo-first^58,59^ and pseudo-second-order^59,60^ kinetic models, and with parabolic diffusion, Ritger and Peppas, modified Freundlich, K-K and Higuchi models.

### Statistical analysis

The difference factor (F1) and similarity factor (F2) were used to compare CBZ release profiles, according to Moore and Flanner^72,73^. The difference factor is a measurement of the relative error between the two curves (Eq. 3).

3 $$F1=\frac{{\sum }_{t=1}^{n}\left|Rt-Tt\right|}{{\sum }_{t=1}^{n}Rt}\times 100$$

where n is the number of time points, Rt is the release value of the reference (pure CBZ) at the time t, and Tt is the release value of the test at time t.

The F2 is a measurement of the similarity in the percent release between the curves (Eq. 4).

4 $$F2=50 \times log\left({\left[1+(1/n)\sum \limits_{t=1}^n {(Rt-Tt)}^{2}\right]}^{-0.5} \times 100\right)$$

For curves to be considered similar, F1 value should be close to 0 (up to 15) and F2 value should be close to 100 (greater than 50).

## Data Availability

No datasets were generated or analyzed during the current study.
